# The study of the traffic deaths in the city of Zarand seven years after the implementation of the safe community (2013-2017)

**Published:** 2019-08

**Authors:** Batoul Rabbani, Ali-Reza Abadi, Maryam Hosseinpour, Ashraf Yazdizadeh

**Affiliations:** ^*a*^Kerman University of Medical Sciences, Kerman, Iran.; ^*b*^Shahid Beheshti University of Medical Sciences Tehran, Iran.

## Abstract

**Background:**

Traffic accidents eliminate huge human potential, and also have significant social and economic consequences. To address this public health problem, the World Health Organization has nominated a Credential safe community. Purposes: The present study was designed to evaluate the effect of the implementation of the safe community of Zarand (7 years after the implementation) on the mortality rate of traffic accidents during 2013-2017.

**Methods:**

This study is a descriptive study. To determine the process of traffic accident deaths seven years after the implementation of the safe community of Zarand (2013-2017) and calculating YLL (Years potential life loss), the information was derived from the Iran Death Register and the life table of Iranian population (life expectancy index from birth). SPSS V24 and Excel software were used to analyze the data.

**Results:**

In this study, in 2015, 2016 and 2017, 6.62, 8 and 5.49% of deaths were due to traffic accidents in Zarand city. The highest deaths from traffic accidents were at the age of 25-64 years. Due to traffic accidents, the person loses his life earlier than 1000 People of Population covered of Kerman University and Zarand Health Center respectively (in year 2017), 8.85 and 8.34 years, respectively.

**Conclusions:**

According to the results of our study, the mortality rate of traffic accidents has varied over these five years. It is recommended to study other indicators of events in order to better judge the effectiveness of the implementation of the safe community plan in Zarand. Key words: Safe community, traffic accidents, mortality, Cause of death and life expectancy.

**Keywords:**

Safe community, Traffic accidents, Mortality, Cause of death and life expectancy

